# Psychiatric gene discoveries shape evidence on ADHD's biology

**DOI:** 10.1038/mp.2015.163

**Published:** 2015-11-17

**Authors:** A Thapar, J Martin, E Mick, A Arias Vásquez, K Langley, S W Scherer, R Schachar, J Crosbie, N Williams, B Franke, J Elia, J Glessner, H Hakonarson, M J Owen, S V Faraone, M C O'Donovan, P Holmans, IMAGE 2 Consortium

**Affiliations:** 1 Institute of Psychological Medicine and Clinical Neurosciences and MRC Centre for Neuropsychiatric Genetics and Genomics, Cardiff University School of Medicine, Cardiff, UK; 2 Department of Quantitative Health Sciences, Department of Psychiatry, University of Massachusetts Medical School, Worcester, MA, USA; 3 Department of Psychiatry, University of Massachusetts Medical School, Worcester, MA, USA; 4 Radboud University Medical Center, Donders Institute for Brain, Cognition and Behaviour, Departments of Psychiatry and Human Genetics, Nijmegen, The Netherlands; 5 Radboud University Medical Center, Donders Institute for Brain, Cognition and Behaviour, Department of Cognitive Neuroscience, Nijmegen, The Netherlands; 6 School of Psychology, Cardiff University, Cardiff, UK; 7 Centre for Applied Genomics, Hospital for Sick Children, Toronto, Ontario, Canada; 8 Department of Psychiatry, Neurosciences and Mental Health, Hospital for Sick Children, University of Toronto, Toronto, Ontario, Canada; 9 Department of Pediatrics and Psychiatry, Sidney Kimmel College of Medicine, Thomas Jefferson University, Philadelphia, PA, USA; 10 Nemours Neuroscience Center, Wilmington, DE, USA; 11 Department of Psychiatry, University of Pennsylvania, Pennsylvania, PA, USA; 12 Center for Human Genetic Research, Massachusetts General Hospital, Boston, MA, USA; 13 Center for Applied Genomics, Children's Hospital of Philadelphia, Philadelphia, PA, USA; 14 Departments of Psychiatry and of Neuroscience and Physiology, SUNY Upstate Medical University, Syracuse, NY, USA; 15 K.G. Jebsen Centre for Research on Neuropsychiatric Disorders, University of Bergen, Bergen, Norway

## Abstract

A strong motivation for undertaking psychiatric gene discovery studies is to provide novel insights into unknown biology. Although attention-deficit hyperactivity disorder (ADHD) is highly heritable, and large, rare copy number variants (CNVs) contribute to risk, little is known about its pathogenesis and it remains commonly misunderstood. We assembled and pooled five ADHD and control CNV data sets from the United Kingdom, Ireland, United States of America, Northern Europe and Canada. Our aim was to test for enrichment of neurodevelopmental gene sets, implicated by recent exome-sequencing studies of (a) schizophrenia and (b) autism as a means of testing the hypothesis that common pathogenic mechanisms underlie ADHD and these other neurodevelopmental disorders. We also undertook hypothesis-free testing of all biological pathways. We observed significant enrichment of individual genes previously found to harbour schizophrenia *de novo* non-synonymous single-nucleotide variants (SNVs; *P*=5.4 × 10^−4^) and targets of the Fragile X mental retardation protein (*P*=0.0018). No enrichment was observed for activity-regulated cytoskeleton-associated protein (*P*=0.23) or *N*-methyl-D-aspartate receptor (*P*=0.74) post-synaptic signalling gene sets previously implicated in schizophrenia. Enrichment of ADHD CNV hits for genes impacted by autism *de novo* SNVs (*P*=0.019 for non-synonymous SNV genes) did not survive Bonferroni correction. Hypothesis-free testing yielded several highly significantly enriched biological pathways, including ion channel pathways. Enrichment findings were robust to multiple testing corrections and to sensitivity analyses that excluded the most significant sample. The findings reveal that CNVs in ADHD converge on biologically meaningful gene clusters, including ones now established as conferring risk of other neurodevelopmental disorders.

## Introduction

Attention-deficit hyperactivity disorder (ADHD), a childhood-onset neurodevelopmental disorder,^1^ is highly heritable.^2, 3^ Despite this strongly consistent finding, some remain sceptical about the diagnosis of ADHD, the biological validity of such a construct, and its neurodevelopmental origins.^4^ Public misunderstanding is further fuelled by the relative scarcity of knowledge regarding its pathophysiology. The knowledge gap on pathophysiology is not straightforward to address given practical limitations to directly assess biological and molecular systems in those who are affected. Genetic findings offer one non-invasive approach to providing clues about the biology and pathogenesis of neuropsychiatric disorders.^5, 6, 7^

In the last 5 years, one class of genetic variant (large, rare chromosomal deletions and duplications (copy number variants; CNVs)) has been found to contribute to ADHD risk across multiple studies.^8, 9, 10, 11, 12^ Common genetic variants also contribute to ADHD when considered ‘en masse' as a composite risk.^13, 14, 15, 16, 17, 18, 19^ One important observation from the original ADHD CNV studies was that the CNVs spanned chromosomal and gene regions that overlapped with schizophrenia and autism CNV loci.^9, 11^ Since then, exome-sequencing investigations of schizophrenia and autism have been published and have highlighted additional novel genomic regions that harbour neurodevelopmental disorder risk variants (single-nucleotide variants; SNVs).^20, 21, 22, 23^ Exome-sequencing results for ADHD are awaited. Unlike CNVs, which generally span multiple genes, SNVs are located in individual genes and thus offer improved resolution for testing cross-disorder pathogenic mechanisms.

Schizophrenia and autism genetic findings have also indicated specific biological mechanisms; these involve targets of the Fragile X Mental Retardation protein (FMRP) in schizophrenia and autism^20, 21, 22, 23, 24^ and in schizophrenia, genes involved in glutamatergic post-synaptic processes: ARC (activity-regulated cytoskeleton-associated protein) and NMDAR (*N*-methyl-D-aspartate receptor) complexes.^20, 21, 25^ Additional pathways have been reported to be enriched for SNVs (for example, calcium channel complexes in schizophrenia^21^), but such findings are not yet replicated across multiple studies and research designs.

In the current study, we assemble five ADHD and control CNV data sets from the United Kingdom, Ireland, Northern Europe, United States of America and Canada.^10^ Our aim was to test the hypothesis that ADHD-associated CNVs show enrichment for gene sets that have been: (a) implicated by SNVs in the most recent, largest schizophrenia and autism *de novo* exome-sequencing studies^20, 21, 22, 23^ and (b) selected for *function* involving ARC, NMDAR and FMRP targets. We also undertook a hypothesis-free meta-analysis of all biological pathways across the five ADHD data sets.

## Materials and methods

### Participant and control data sets

The recruitment, assessment processes and clinical description of ADHD case subjects and controls have been described in detail previously.^10^ Case–-control CNV data sets were provided by the CNV lead for each study (SS, NW, JG, BF, EM). The five data sets came from: (a) Canada,^11^ (b) Cardiff, UK,^9, 17^ (c) the Children's Hospital of Philadelphia, USA (CHOP),^8^ (d) the International Multi-Center ADHD Genetics (IMAGE) 2 Project^10, 16^ and (e) the Pfizer-funded study from UCLA, Washington University and Massachusetts General Hospital (PUWMa).^26^ All affected subjects from each of the five studies were children aged 5–18 years, had an IQ⩾70, were of European descent, free of psychosis, epilepsy, serious neurological impairment and met Diagnostic and Statistical Manual of Mental Disorders (DSM)-III-R or DSM-IV criteria for ADHD as confirmed by semi-structured research diagnostic interviews. Collection and analysis of case–control data had been approved at each site by each Institutional Review Board or ethics committee. Case data were collected with informed consent from parents and assent from children.

After quality control exclusions (see later), the Canadian study included 247 DSM-IV ADHD cases and 2357 comparison subjects genotyped on the Affymetrix 6.0 array (Affymetrix, Santa Clara, CA, USA). The UK Cardiff study (excluding those that were included in IMAGE 2) included 603 DSM-IV ADHD cases genotyped on Illumina Human 660Q-Quad Beadchip (Illumina, San Diego, CA, USA) and 1047 comparison subjects genotyped on HumanHap550Beadchip. The CHOP study included 1013 participants with DSM-IV ADHD and 4105 comparison children genotyped on the Illumina Infinium HumanHap550K Beadchip. IMAGE 2 included data from 732 affected children collected in the United Kingdom, Ireland, Germany, Switzerland, the Netherlands and the United States. Control data came from 2010 subjects collected for a genome-wide association study of schizophrenia, described elsewhere.^27^ A total of 692 ADHD cases from PUWMa and 1101 controls were genotyped on the Illumina 1M BeadChip.

Quality control and procedures for CNV calling are described in each of the original manuscripts (see Williams *et al.*^10^ for a summary). CNVs were filtered for frequency (<1%), and also for overlapping (>50% length) sites of known common CNVs defined by the Genome Structural Variation Consortium (http://projects.tcag.ca/variation/ng42m_cnv.php) or segmental duplications present in the March, 2006, human reference sequence (National Center for Biotechnology Information reference build 36.1, hg18). To allow for differences in CNV detection between the arrays, we used controls genotyped on platforms that matched the platforms used for the cases. The primary analyses used all CNVs >500 kb as these are the most reliably called across different genotyping platforms, have consistently been found to show increased burden in ADHD cases vs controls and have withstood experimental validation.^10^ Pathways highlighted by the primary analysis were also tested for enrichment with all CNVs >100 kb (results available from last author).

### Statistical method for enrichment testing

The method is adapted from that proposed by Raychaudhuri *et al.*^28^ and has been successfully applied to *de novo* schizophrenia CNVs.^25^ Briefly, each CNV was labelled ‘case/control' according to whether it occurs in a case or a control. A ‘study' covariate for each data set was included as this corrects for variation in the genotyping assays and CNV estimation algorithms used across the different studies.

The following two logistic regression models were fitted to the sample of CNVs:

1. Case–control study+CNV length+number of genes hit outside pathway
2. Case–control study+CNV length+number of genes hit outside pathway+hit gene in pathway (yes/no)

and the deviances of the two models compared. If there is no enrichment of case CNV hits on pathway genes, then the difference in deviances should be distributed asymptotically as a *χ*^2^ on one degree of freedom.^29^ CNV length was fitted in the model because long CNVs are more likely to hit any set of genes than small ones, and CNV length may differ systematically between cases and controls. The ‘number of genes hit outside pathway' was fitted to allow for case CNVs influencing disease status by hitting genes other than those in the pathway being tested. A binary variable (yes/no) is used for whether a CNV ‘hits gene(s) in a pathway' rather than the number of genes in the pathway hit by the CNV (which is also a possibility) to allow for some pathways having several genes that are physically close together (thus, likely to be hit by the same CNV). The same analysis method was used to determine gene-specific enrichments, by defining the ‘pathway' as the gene.

For all analyses, we tested pathways showing significant enrichment in the combined data set of five studies and then undertook tests of sensitivity by examining each of the five data sets separately and rerunning analysis after excluding the most significant single sample. Additional analyses were stratified by CNV type (deletions and duplications). As the number of duplications was greater than the number of deletions,^10^ we tested for differential strengths of enrichment by comparing case duplications to case deletions in the logistic regression framework described above.

### Specific gene sets

#### Genes selected by location

Four sets of genes were defined: (a) non-synonymous and (b) loss-of-function *de novo* SNVs in schizophrenia were taken from the most recent published *de novo* exome-sequencing study of schizophrenia, which also catalogued and annotated all SNVs in previous studies in a consistent way.^20^ There were a total of 611 genes containing at least one non-synonymous *de novo* SNV, with 87 of these containing a *de novo* loss-of-function SNV. We also took sets of genes containing: (c) non-synonymous and (d) loss-of-function *de novo* SNVs from the two largest, recent exome-sequencing studies of autism.^22, 23^ Combining the autism SNV sets from these studies, there were 2726 unique genes containing at least one non-synonymous *de novo* SNV, of which 538 contained a loss-of-function *de novo* SNV.

#### Genes selected by function: FMRP targets, ARC and NMDAR

Next, we examined a further three gene sets by function, selecting those implicated in schizophrenia and autism by CNV and more recent exome-sequencing studies. These were genes involved in FMRP targets (840 genes) as defined previously^30^ and the ARC (28 genes) and NMDAR (61 genes) complexes, as defined by Kirov *et al.*^25^ Bonferroni corrections were used to adjust for testing seven gene sets.

#### Hypothesis-free analysis of all pathways

This final analysis was undertaken using a large, unbiased, general set of pathways comprising:

- Gene Ontology^31^ (http://www.geneontology.org/, accessed on July 2013)
- KEGG^32^ (http://www.genome.jp/kegg/, accessed on June 2013)
- PANTHER pathways version 3.1 (http://www.pantherdb.org/pathway/)
- Mouse Genome Informatics database^33^ (http://www.informatics.jax.org/, accessed on August 2013)
- BioCarta (http://www.biocarta.com, accessed on June 2013)
- Reactome^34^ (http://www.reactome.org, accessed on June 2013)
- NCI^35^ (http://pid.nci.nih.gov, accessed on June 2013)

Pathways containing between 3 and 1500 genes were used in the analysis (15 111 in total). To increase the accuracy of the asymptotic *P*-values described above and to reduce the chance of a small pathway being falsely declared to be enriched based on a few gene hits, analysis was restricted to pathways with at least 10 gene hits in the total sample (7034 in total). Correction for multiple testing of pathways was performed by calculating *q*-values.^36^

## Results

Table 1 shows the number of large, rare CNVs (>500 kb) for each of the previously published five case control samples and the total number of deletions and duplications. The rate and burden of CNVs for each sample have been published previously.^9, 10, 11, 26, 37^

### Gene sets selected by location of schizophrenia and autism *de novo* SNVs

The genes containing non-synonymous schizophrenia *de novo* SNVs^20^ were significantly enriched for case ADHD CNV hits (*P*=5.4 × 10^−4^ for CNVs >500 kb, see Table 2), which is significant after Bonferroni correction. Findings remained significant even after the most significant single sample was removed from the analysis (Table 2). The significant enrichment was observed for duplications (*P*=5.6 × 10^−4^) but not for deletions (*P*=0.37). However, there was no significant difference between ADHD duplication and deletions in terms of the rate of hits for genes previously found to carry non-synonymous schizophrenia SNVs (*P*=0.142). For sample-specific enrichments, see Supplementary Table 1, and see Supplementary Table 2 for gene-wide *P*-values. Restricting analysis to genes hit by loss-of-function *de novo* schizophrenia SNVs showed no significant enrichment for case CNV hits.

Table 2 shows a nominally significant enrichment of CNVs among autism *de novo* SNV genes (non-synonymous or loss-of-function), although these do not survive Bonferroni correction.

### Gene sets selected by function

There was significant enrichment of ADHD case CNVs in FMRP targets (*P*=0.0018), which remained significant after Bonferroni correction (see Table 3 for details). The enrichments also remained significant when the most significant single sample was removed. Significant enrichment was observed for duplications (*P*=0.005) but not for deletions (*P*=0.18), although there was no significant difference between the rate of case duplication and deletion hits for FMRP target genes (*P*=0.247). See Supplementary Table 3 for sample-specific enrichments and Supplementary Table 4 for FMRP target genes that showed significant enrichment. There was no evidence of enrichment of ARC complex or NMDAR gene sets.

### Hypothesis-free analysis of all pathways

The most significantly enriched pathways are shown in Table 4, with those remaining significant in the sensitivity analysis highlighted in bold. As can be seen from the *q*-values, many of these were highly significant even after correction for multiple testing of pathways. Much of the enrichment appeared to come from duplications, with the exception of the ion channel pathways where enrichment was observed in both deletions and duplications but the strength of enrichment did not differ significantly for duplications and deletions (see Supplementary Table 5 for details). Ion channel pathways (ligand gated ion channel activity and ion gated channel activity), transmembrane transport and organonitrogen compound catabolic process were robust to sensitivity analysis (see Supplementary Table 6 for enrichment effect sizes and the number of gene hits for pathways listed in Table 4; see Supplementary Tables 7-9 for most significant ion channel pathway genes, organonitrogen compound catabolic process and carbohydrate derivative catabolic process genes and transmembrane transport genes).

Secondary analyses of CNVs >100 kb showed that observed pathways were generally significantly enriched in these analysis (results available from last author).

## Discussion

In these analyses of ADHD case–control CNV data, the largest to date, we found highly significant enrichment of CNVs in many, although not all, hypothesised neurodevelopmental gene sets. Hypothesis-free testing revealed additional biological pathways enriched for CNVs in those with ADHD. Genes spanned by the ADHD CNVs were enriched for those that have recently been found to harbour schizophrenia-associated *de novo* SNVs. Previous work demonstrated overlap of ADHD CNVs with schizophrenia and autism CNVs;^9, 10, 11^ however, because we were now able to define our gene sets using SNVs, which impact on single genes rather than chromosomal regions encompassing multiple genes, the current findings more precisely suggest overlap at the level of genes. These findings therefore extend, refine and are independent of previous studies that suggest biological overlap across these clinically very different disorders.

The clinical co-morbidity and co-heritability of ADHD is much more strongly established with autism than with schizophrenia.^38^ Although previous studies, using some of the data sets in the present paper, found ADHD and autism overlap at the level of CNVs^9, 10, 11^ and CNV-associated biological pathways,^39^ we did not observe this extended to autism *de novo* SNV genes.

Targets of the FMRP have been implicated previously in both schizophrenia and autism.^21, 22, 23^ Complete expression failure of FMRP itself, which characterises Fragile X syndrome, is known to be associated with elevated rates of ADHD, autism and other neurodevelopmental disorders.^40^ Indeed, the majority of males with Fragile X syndrome show ADHD. Previous work has shown that the protein FMRP regulates activity of 842 biological targets and suggested that some of these likely underlie the manifestation of autistic features in Fragile X syndrome.^30^ Our findings show that genes encoding FMRP targets are also enriched in ADHD CNVs, which could help explain why children with Fragile X syndrome show such high rates of ADHD. The results suggest that FMRP-mediated biology may be relevant across multiple neuropsychiatric disorders that include ADHD, as well as autism and schizophrenia. Although a recent report suggests that what has been considered as the specific contribution of FMRP targets to the pathogenesis of autism might simply reflect involvement of long, highly brain-expressed genes,^41^ here large gene size is controlled for. Nevertheless, further work is needed to understand how FMR1 is involved in the pathogenesis of different neurodevelopmental phenotypes, that is, ADHD, autism and schizophrenia, each of which has very different phenotype features and treatments.

Although schizophrenia and autism genetic findings have all strongly implicated the involvement of synaptic functions in pathogenesis,^20, 21, 23^ we did not observe that to be the case for ADHD. Nor did we replicate prior pathway analysis implicating neurite outgrowth at synapses in ADHD, although previous analyses have used SNP or candidate gene data or smaller data sets.^42, 43, 44, 45^ We cannot be certain if this is a genuine point of difference or whether larger studies of ADHD, which consider additional types of mutations, for example, through exome sequencing, will yield a different pattern of findings.

The hypothesis-free analysis of all ADHD CNV pathways yielded strongly significant enrichment for multiple biological pathways. Many but not all of these were robust to analysis after excluding the most significant study. As expected for this final, hypothesis-free set of analyses, the significance of the enrichment was reduced by the removal of the most significant study, even when there was no significant difference in the strength of the enrichment between that study and the remainder of the sample, as was the case for most of the pathways highlighted here (see Supplementary Tables 1 for details). Thus, this analysis should be regarded as a sensitivity analysis testing the robustness of the pathway enrichment, rather than a measure of its overall significance. The fact that the enrichment strengths were not significantly different for most of the pathways of interest gives further evidence for consistency of enrichment across studies.

Ion channel pathways (ligand gated ion channel activity and ion gated channel activity) and especially involvement of *CHRNA7* and associated pathways have been implicated in previous analyses of a subset of the present pooled analysis.^10, 17, 39^ Although some enrichment of immune-related pathways was also observed in the current study, this finding was not robust to the sensitivity analysis and thus requires cautious interpretation until more data become available. Regardless, the present study demonstrates that most findings were robust to sensitivity testing, whereby we excluded the most significant sample, despite potential variation in case mix severity and ascertainment across research centres and concerns from some quarters about the validity of ADHD.

Although our study involves analysis of the largest ADHD CNV data set to date, there are several limitations. First, analyses of this type for any disorder are restricted by the quality of gene and pathway annotations and the fact that the same genes belong to multiple different functional gene sets. Second, genotyped ADHD sample sizes have markedly lagged behind those of many other neurodevelopmental and psychiatric disorders and results of exome sequencing are awaited. Third, ADHD-associated CNVs do not capture all possible forms of genetic risk, or risk loci; however, previous findings from smaller, individual studies do suggest biological convergence of CNVs and common gene variants.^7, 17, 42, 44, 45^ However, the biology and validity of ADHD remain poorly understood and it is still widely considered to be primarily a catecholaminergic disorder.^43^ Findings from the present study highlight the consistent and robust neurodevelopmental nature of ADHD and provide novel insights about its biological underpinnings.

In conclusion, in an international, cross-centre analysis of ADHD CNV data, we find evidence of biological overlap with schizophrenia at multiple levels; previous findings of overlap with schizophrenia CNVs have now been extended to SNVs. Furthermore, FMRP target enrichment appears to characterise ADHD as well as schizophrenia and autism. Ion channel pathway involvement in ADHD was robust to type of CNV and across samples. The findings reveal that CNVs in children with ADHD, from across multiple centres, converge on biologically meaningful gene clusters that are now robustly established as involved in neurodevelopmental disorder risk.

## Figures and Tables

**Table 1:** Burden and type of CNVs >500 kb in cases and controls from each study

*CNV sample*	*CNVs*	*Number of CNVs*				*Number of deletions*				*Number of duplications*			
		*ADHD cases*	*Controls*	*Ratio*	P	*ADHD cases*	*Controls*	*Ratio*	P	*ADHD cases*	*Controls*	*Ratio*	P
Canada^11^	*N*	22	217	0.97	0.88	4	52	0.73	0.55	18	165	1.04	0.87
	Rate	0.089	0.092			0.016	0.022			0.073	0.070		
Cardiff^9, 17^	*N*	65	78	1.45	0.021	13	13	1.74	0.15	52	65	1.39	0.066
	Rate	0.108	0.074			0.022	0.012			0.086	0.062		
CHOP^8^	*N*	220	382	2.33	4.7 × 10^−28^	28	80	1.42	0.11	192	302	2.58	4.4 × 10^−^^29^
	Rate	0.217	0.093			0.028	0.019			0.190	0.074		
IMAGE 2 (refs 10, 16)	*N*	89	191	1.28	0.042	22	47	1.29	0.32	67	144	1.28	0.084
	Rate	0.122	0.095			0.030	0.023			0.092	0.072		
PUWMa^26^	*N*	59	86	1.09	0.59	25	28	1.42	0.19	34	58	0.93	0.74
	Rate	0.085	0.078			0.036	0.025			0.049	0.053		

Abbreviations: ADHD, attention-deficit hyperactivity disorder; CHOP, Children's Hospital of Philadelphia; CNV, copy number variant; IMAGE, International Multi-Center ADHD Genetics; PUWMa, Pfizer-funded study from UCLA, Washington University and Massachusetts General Hospital.

*N*: the number of CNVs observed in the sample; rate: the average number of CNVs per person.

**Table 2:** Enrichment of ADHD case CNV hits in genes containing schizophrenia and autism *de novo* SNVs (non-synonymous and loss-of-function)

*Gene set*	a*Genes*	*CNVs >500 kb*			
		P	P *(minus best)*^b^	P *(del)*	P *(dup)*
SCZ (NS)	611	5.4 × 10^−4^	0.015	0.37	5.6 × 10^−4^
SCZ (LoF)	87	0.33	0.40	0.50	0.27
AUT (NS)	2726	0.019	0.054	0.31	0.024
AUT (LoF)	538	0.026	0.54	0.16	0.072

Abbreviations: ADHD, attention-deficit hyperactivity disorder; AUT, autism; CNV, copy number variant; Del, deletion; Dup, duplication; LoF, loss of function; NS, non-synonymous; SCZ, schizophrenia; SNV, single-nucleotide variant.

a Number.

b *P*-value for pathway enrichment after omitting the most significant sample.

**Table 3:** Enrichment of FMRP, ARC and NMDAR gene sets for ADHD case CNV hits

*Gene set*	*# Genes*	*CNVs >500 kb*			
		P	P *(minus best)*^a^	P *(del)*	P *(dup)*
FMRP	840	0.0018	0.032	0.18	0.0050
ARC	28	0.23	0.44	0.12	0.40
NMDAR	61	0.74	0.94	0.92	0.55

Abbreviations: ADHD, attention-deficit hyperactivity disorder; ARC, activity-regulated cytoskeleton-associated protein; CNV, copy number variant; Del, deletion; Dup, duplication; FMRP, Fragile X mental retardation protein; NMDAR, *N*-methyl-D-aspartate receptor.

a *P*-value for pathway enrichment after omitting the most significant sample.

**Table 4:** Top pathways in pooled meta-analysis with most significant enrichment for ADHD case CNV hits among CNVs >500 kb

*Pathway name*	*Pathway ID*	*# Genes*	P	q*-value*	P *(minus best)*^a^
IL6-mediated signalling events	NCI:62	82	1.33 × 10^−11^	9.17 × 10^−8^	0.256
TGF-β receptor signalling	NCI:95	44	4.90 × 10^−11^	1.69 × 10^−7^	0.508
Defence response to virus	GO:51607	147	7.02 × 10^−8^	1.62 × 10^−4^	0.31
Respiratory electron transport	**REACT:1019**	**81**	**1.98 × 10**^**−7**^	**3.41 × 10**^**−4**^	**0.02**
Organonitrogen compound catabolic process	**GO:1901565**	**893**	**9.08 × 10**^**−7**^	**1.05 × 10**^**−3**^	**3.13 × 10**^**−5**^
Transmembrane transporter activity	**GO:22857**	**902**	**9.10 × 10**^**−7**^	**1.05 × 10**^**−3**^	**8.85 × 10**^**−4**^
Citric acid (TCA) cycle and respiratory electron transport	REACT:1240	118	1.16 × 10^−6^	1.15 × 10^−3^	0.057
Carbohydrate derivative catabolic process	**GO:1901136**	**747**	**2.19 × 10**^**−6**^	**1.61 × 10**^**−3**^	**3.34 × 10**^**−4**^
Ligand-gated ion channel activity	**GO:15276**	**136**	**2.33 × 10**^**−6**^	**1.61 × 10**^**−3**^	**3.41 × 10**^**−4**^
Methyltransferase activity	**GO:8168**	**201**	**3.19 × 10**^**−6**^	**2.00 × 10**^**−3**^	**0.022**
Small thymus	MGI:706	199	4.15−10^−6^	2.39 × 10^−3^	0.488
Transmembrane transport	**GO:55085**	**1124**	**5.22 × 10**^**−6**^	**2.44 × 10**^**−3**^	**0.0028**
Ion-gated channel activity	**GO:22839**	**300**	**5.29 × 10**^**−6**^	**2.44 × 10**^**−3**^	**4.18 × 10**^**−4**^

Abbreviations: ADHD, attention-deficit hyperactivity disorder; CNV, copy number variant; GO, Gene Ontology; IL, interleukin; MGI, Mouse Genome Informatics; TCA, tricarboxylic acid; TGF, transforming growth factor.

Pathways that are robustly enriched (that is, those with *P* (minus best)<0.05) are shown in bold.

a *P*-value for pathway enrichment after omitting the most significant sample.

## References

[bib1] Sonuga-Barke EJS, Taylor E. ADHD and Hyperkinetic Disorder. In: Thapar A, Pine D, Leckman JF, Scott S, Snowling MJ, Taylor E (eds). Rutter's Child and Adolescent Psychiatry, 6th (edn). John Wiley and Sons Limited: Chichester, UK, 2015.

[bib2] Thapar A, Cooper M, Eyre O, Langley K. What have we learnt about the causes of ADHD? J Child Psychol Psychiatry 2013; 54: 3–16. 22963644 10.1111/j.1469-7610.2012.02611.x PMC3572580

[bib3] Faraone SV, Mick E. Molecular genetics of attention deficit hyperactivity disorder. Psychiatr Clin North Am 2010; 33: 159–180. 20159345 10.1016/j.psc.2009.12.004 PMC2847260

[bib4] Timimi S, Taylor E. ADHD is best understood as a cultural construct. Br J Psychiatry 2003; 184: 8–9. 10.1192/bjp.184.1.8 14702221

[bib5] The Network and Pathway Analysis Subgroup of the Psychiatric Genomics Consortium. Psychiatric genome-wide association study analyses implicate neuronal, immune and histone pathways. Nat Neurosci 2015; 18: 199–209. 25599223 10.1038/nn.3922 PMC4378867

[bib6] Nurnberger JI, Koller DL, Jung J, Edenberg HJ, Foroud T, Guella I et al. Identification of pathways for bipolar disorder: a meta-analysis. JAMA Psychiatry 2014; 71: 657–664. 24718920 10.1001/jamapsychiatry.2014.176 PMC4523227

[bib7] Hawi Z, Cummins TDR, Tong J, Johnson B, Lau R, Samarrai W et al. The molecular genetic architecture of attention deficit hyperactivity disorder. Mol Psychiatry 2015; 20: 289–297. 25600112 10.1038/mp.2014.183

[bib8] Elia J, Glessner JT, Wang K, Takahashi N, Shtir CJ, Hadley D et al. Genome-wide copy number variation study associates metabotropic glutamate receptor gene networks with attention deficit hyperactivity disorder. Nat Genet 2012; 44: 78–84. 10.1038/ng.1013 PMC4310555 22138692

[bib9] Williams NM, Zaharieva I, Martin A, Langley K, Mantripragada K, Fossdal R et al. Rare chromosomal deletions and duplications in attention-deficit hyperactivity disorder: a genome-wide analysis. Lancet 2010; 376: 1401–1408. 20888040 10.1016/S0140-6736(10)61109-9 PMC2965350

[bib10] Williams NM, Franke B, Mick E, Anney RJL, Freitag CM, Gill M et al. Genome-wide analysis of copy number variants in attention deficit hyperactivity disorder: the role of rare variants and duplications at 15q13. 3. Am J Psychiatry 2012; 169: 195–204. 22420048 10.1176/appi.ajp.2011.11060822 PMC3601405

[bib11] Lionel AC, Crosbie J, Barbosa N, Goodale T, Thiruvahindrapuram B, Rickaby J et al. Rare copy number variation discovery and cross-disorder comparisons identify risk genes for ADHD. Sci Transl Med 2011; 3: 95ra75. 10.1126/scitranslmed.3002464 21832240

[bib12] Jarick I, Volckmar AL, Pütter C, Pechlivanis S, Nguyen TT, Dauvermann MR et al. Genome-wide analysis of rare copy number variations reveals PARK2 as a candidate gene for attention-deficit/hyperactivity disorder. Mol Psychiatry 2012; 19: 115–121. 23164820 10.1038/mp.2012.161 PMC3873032

[bib13] Lee SH, Ripke S, Neale BM, Faraone SV, Purcell SM, Perlis RH et al. Genetic relationship between five psychiatric disorders estimated from genome-wide SNPs. Nat Genet 2013; 45: 984–994. 23933821 10.1038/ng.2711 PMC3800159

[bib14] Hamshere ML, Langley K, Martin J, Agha SS, Stergiakouli E, Anney RJ et al. High loading of polygenic risk for ADHD in children with comorbid aggression. Am J Psychiatry 2013; 170: 909–916. 23599091 10.1176/appi.ajp.2013.12081129 PMC3935265

[bib15] Yang L, Neale BM, Liu L, Lee SH, Wray NR, Ji N et al. Polygenic transmission and complex neuro developmental network for attention deficit hyperactivity disorder: genome-wide association study of both common and rare variants. Am J Med Genet Part B Neuropsychiatr Genet 2013; 162B: 419–430. 10.1002/ajmg.b.32169 PMC4321789 23728934

[bib16] Neale BM, Medland SE, Ripke S, Asherson P, Franke B, Lesch K-PP et al. Meta-analysis of genome-wide association studies of attention-deficit/hyperactivity disorder. J Am Acad Child Adolesc Psychiatry 2010; 49: 884–897. 20732625 10.1016/j.jaac.2010.06.008 PMC2928252

[bib17] Stergiakouli E, Hamshere M, Holmans P, Langley K, Zaharieva I, Hawi Z et al. Investigating the contribution of common genetic variants to the risk and pathogenesis of ADHD. Am J Psychiatry 2012; 169: 186–194. 22420046 10.1176/appi.ajp.2011.11040551 PMC3601404

[bib18] Martin J, Hamshere ML, Stergiakouli E, O'Donovan MC, Thapar A. Genetic risk for attention-deficit/hyperactivity disorder contributes to neurodevelopmental traits in the general population. Biol Psychiatry 2014; 76: 664–671. 24673882 10.1016/j.biopsych.2014.02.013 PMC4183378

[bib19] Groen-Blokhuis MM, Middeldorp CM, Kan K-J, Abdellaoui A, Beijsterveldt CEM van, Ehli EA et al. Attention deficit hyperactivity disorder polygenic risk scores predict attention problems in a population-based sample of children. J Am Acad Child Adolesc Psychiatry 2014; 53: 1123–1129. 25245356 10.1016/j.jaac.2014.06.014

[bib20] Fromer M, Pocklington AJ, Kavanagh DH, Williams HJ, Dwyer S, Gormley P et al. *De novo* mutations in schizophrenia implicate synaptic networks. Nature 2014; 506: 179–184. 24463507 10.1038/nature12929 PMC4237002

[bib21] Purcell SM, Moran JL, Fromer M, Ruderfer D, Solovieff N, Roussos P et al. A polygenic burden of rare disruptive mutations in schizophrenia. Nature 2014; 506: 185–190. 24463508 10.1038/nature12975 PMC4136494

[bib22] Iossifov I, O'Roak BJ, Sanders SJ, Ronemus M, Krumm N, Levy D et al. The contribution of de novo coding mutations to autism spectrum disorder. Nature 2014; 515: 216–221. 25363768 10.1038/nature13908 PMC4313871

[bib23] De Rubeis S, He X, Goldberg AP, Poultney CS, Samocha K, Ercument Cicek A et al. Synaptic, transcriptional and chromatin genes disrupted in autism. Nature 2014; 515: 209–215. 25363760 10.1038/nature13772 PMC4402723

[bib24] Szatkiewicz JP, O'Dushlaine C, Chen G, Chambert K, Moran JL, Neale BM et al. Copy number variation in schizophrenia in Sweden. Mol Psychiatry 2014; 19: 762–773. 24776740 10.1038/mp.2014.40 PMC4271733

[bib25] Kirov G, Pocklington AJ, Holmans P, Ivanov D, Ikeda M, Ruderfer D et al. *De novo* CNV analysis implicates specific abnormalities of postsynaptic signalling complexes in the pathogenesis of schizophrenia. Mol Psychiatry 2011; 17: 142–153. 22083728 10.1038/mp.2011.154 PMC3603134

[bib26] Mick E, Todorov A, Smalley S, Hu X, Loo S, Todd RD et al. Family-based genome-wide association scan of attention-deficit/hyperactivity disorder. J Am Acad Child Adolesc Psychiatry 2010; 49: 898–905. 20732626 10.1016/j.jaac.2010.02.014 PMC3730251

[bib27] Korn JM, Kuruvilla FG, McCarroll SA, Wysoker A, Nemesh J, Cawley S et al. Integrated genotype calling and association analysis of SNPs, common copy number polymorphisms and rare CNVs. Nat Genet 2008; 40: 1253–1260. 18776909 10.1038/ng.237 PMC2756534

[bib28] Raychaudhuri S, Korn JM, McCarroll SA, Altshuler D, Sklar P, Purcell S et al. Accurately assessing the risk of schizophrenia conferred by rare copy-number variation affecting genes with brain function. PLoS Genet 2010; 6: e1001097. 20838587 10.1371/journal.pgen.1001097 PMC2936523

[bib29] Collett D. Modelling Binary Data. 2nd (edn). Chapman and Hall/CRC Press: London, UK, 2002.

[bib30] Darnell JC, Van Driesche SJ, Zhang C, Hung KYS, Mele A, Fraser CE et al. FMRP stalls ribosomal translocation on mRNAs linked to synaptic function and autism. Cell 2011; 146: 247–261. 21784246 10.1016/j.cell.2011.06.013 PMC3232425

[bib31] Harris MA, Clark J, Ireland A, Lomax J, Ashburner M, Foulger R et al. The Gene Ontology (GO) database and informatics resource. Nucleic Acids Res 2004; 32: D258. 14681407 10.1093/nar/gkh036 PMC308770

[bib32] Kanehisa M, Goto S, Sato Y, Furumichi M, Tanabe M. KEGG for integration and interpretation of large-scale molecular data sets. Nucleic Acids Res 2012; 40: D109–D114. 22080510 10.1093/nar/gkr988 PMC3245020

[bib33] Bult CJ, Eppig JT, Kadin JA, Richardson JE, Blake JA. The Mouse Genome Database (MGD): mouse biology and model systems. Nucleic Acids Res 2008; 36: D724. 18158299 10.1093/nar/gkm961 PMC2238849

[bib34] Croft D, O'Kelly G, Wu G, Haw R, Gillespie M, Matthews L et al. Reactome: a database of reactions, pathways and biological processes. Nucleic Acids Res 2011; 39: D691–D697. 21067998 10.1093/nar/gkq1018 PMC3013646

[bib35] Schaefer CF, Anthony K, Krupa S, Buchoff J, Day M, Hannay T et al. PID: the Pathway Interaction Database. Nucleic Acids Res 2009; 37: D674–D679. 18832364 10.1093/nar/gkn653 PMC2686461

[bib36] Storey JD, Tibshirani R. Statistical significance for genomewide studies. Proc Natl Acad Sci USA 2003; 100: 9440–9445. 12883005 10.1073/pnas.1530509100 PMC170937

[bib37] Elia J, Gai X, Xie HM, Perin JC, Geiger E, Glessner JT et al. Rare structural variants found in attention-deficit hyperactivity disorder are preferentially associated with neurodevelopmental genes. Mol Psychiatry 2010; 15: 637–646. 19546859 10.1038/mp.2009.57 PMC2877197

[bib38] Lichtenstein P, Carlström E, Råstam M, Gillberg C, Anckarsäter H. The genetics of autism spectrum disorders and related neuropsychiatric disorders in childhood. Am J Psychiatry 2010; 167: 1357–1363. 20686188 10.1176/appi.ajp.2010.10020223

[bib39] Martin J, Cooper M, Hamshere ML, Pocklington A, Scherer SW, Kent L et al. Biological Overlap of Attention-Deficit/Hyperactivity Disorder and Autism Spectrum Disorder: Evidence From Copy Number Variants. J Am Acad Child Adolesc Psychiatry 2014; 53: 761–770. 24954825 10.1016/j.jaac.2014.03.004 PMC4074351

[bib40] Lozano R, Rosero CA, Hagerman RJ. Fragile X spectrum disorders. Intractable Rare Dis Res 2014; 3: 134–146. 25606363 10.5582/irdr.2014.01022 PMC4298643

[bib41] Ouwenga RL, Dougherty J. Fmrp targets or not: long, highly brain-expressed genes tend to be implicated in autism and brain disorders. Mol Autism 2015; 6: 16. 25789151 10.1186/s13229-015-0008-1 PMC4363463

[bib42] Poelmans G, Pauls DL, Buitelaar JK, Franke B. Integrated genome-wide association study findings: identification of a neurodevelopmental network for attention deficit hyperactivity disorder. Am J Psychiatry 2011; 168: 365–377. 21324949 10.1176/appi.ajp.2010.10070948

[bib43] Bralten J, Franke B, Waldman I, Rommelse N, Hartman C, Asherson P et al. Candidate genetic pathways for attention-deficit/hyperactivity disorder (ADHD) show association to hyperactive/impulsive symptoms in children with ADHD. J Am Acad Child Adolesc Psychiatry 2013; 52: 1204–1212. 24157394 10.1016/j.jaac.2013.08.020

[bib44] Cristino AS, Williams SM, Hawi Z, An JY, Bellgrove MA, Schwartz CE et al. Neurodevelopmental and neuropsychiatric disorders represent an interconnected molecular system. Mol Psychiatry 2014; 19: 294–301. 23439483 10.1038/mp.2013.16

[bib45] Yang L, Neale BM, Liu L, Lee SH, Wray NR, Ji N. Polygenic transmission and complex neuro developmental network for attention deficit hyperactivity disorder: genome-wide association study of both common and rare variants. Am J Med Genet B Neuropsychiatr Genet 2013; 162B: 419–430. 23728934 10.1002/ajmg.b.32169 PMC4321789

