# Supplementary material for: Psychiatric gene discoveries shape evidence on ADHD's biology
Source: Mol Psychiatry. 2015 Nov 17;21(9):1202–7. doi: 10.1038/mp.2015.163 (PMC4820035; doi:10.1038/mp.2015.163)
Supplement: Supplementary Tables [file mp2015163x1.doc]

# Supplementary Materials

**Supplementary Table 1:** Sample-specific enrichments for case CNVs hitting genes containing a non-synonymous schizophrenia SNV

| **CNV set** | **All CNVs >500kb** | **Dup >500kb** | **Del >500kb** |
| --- | --- | --- | --- |
| **p(meta)** | 5.4x10^-4^ | 5.6x10^-4^ | 0.37 |
| **p(CHOP)** | 0.37 | 0.11 | 0.99 |
| **p(Canada)** | 0.075 | 0.13 | 0.13 |
| **p(Cardiff)** | 0.033 | 0.050 | 0.27 |
| **p(IMAGE)** | 0.30 | 0.51 | 0.13 |
| **p(PuWMA)** | 0.011 | 3.5x10^-4^ | 0.88 |
| **p(minusbest)*** | 0.015 | 0.014 | 0.61 |
| ***p (best vs remainder)***** | *0.097* | *0.018* | *0.958* |

* p-value for pathway enrichment after omitting the most significant sample

***p-value for testing difference in effect sizes between the most significant sample and the remainder of the dataset*

SNV: single nucleotide variant; Del: deletion; Dup: Duplication

**Supplementary Table 2:** Gene-wide p-values for genes significantly enriched for both schizophrenia non-synonymous *de novo* SNV and ADHD case CNV (>500kb) hits

| **Gene** | **Chr** | **Position (Mb)** | **p** |
| --- | --- | --- | --- |
| **MIF** | 22 | 24.2 | 0.008 |
| **RYR3** | 15 | 33.6 | 0.010 |
| **ADAMTS1** | 21 | 28.2 | 0.013 |
| **ZNF518B** | 4 | 10.4 | 0.015 |
| **PRDX6** | 1 | 173.4 | 0.022 |
| **KLHL20** | 1 | 173.7 | 0.022 |
| **SLC19A2** | 1 | 169.4 | 0.025 |
| **IFNAR2** | 21 | 34.6 | 0.028 |
| **NRIP1** | 21 | 16.3 | 0.035 |
| **EGFL7** | 9 | 139.6 | 0.049 |

SNV: single nucleotide variant; Chr: chromosome

**Supplementary Table 3:** Sample-specific enrichments for case CNVs hitting FMRP target genes

| **CNV set** | **All CNVs >500kb** | **Dup >500kb** | **Del >500kb** |
| --- | --- | --- | --- |
| **p(meta)** | 0.0018 | 0.0050 | 0.18 |
| **p(CHOP)** | 0.066 | 0.054 | 0.60 |
| **p(Canada)** | 0.10 | 0.14 | 0.80 |
| **p(Cardiff)** | 0.12 | 0.052 | 0.85 |
| **p(IMAGE)** | 0.68 | 0.70 | 0.52 |
| **p(PuWMA)** | 0.32 | 0.42 | 0.24 |
| **p(minusbest)*** | 0.032 | 0.015 | 0.40 |
| **p(best vs remainder)**** | 0.737 | 0.550 | 0.513 |

* p-value for pathway enrichment after omitting the most significant sample

**p-value for testing difference in effect sizes between the most significant sample and the remainder of the dataset

Del: deletion; Dup: Duplication

**Supplementary Table 4:** FMRP target genes showing significant enrichment (p<0.01) for hits by case CNVs >500kb

| **Gene** | **Chr** | **Position (Mb)** | **p** |
| --- | --- | --- | --- |
| **DSCAM** | 21 | 40.3 | 0.0030 |
| **TTC3** | 21 | 37.4 | 0.0016 |
| **APP** | 21 | 26.2 | 0.019 |
| **C9orf172** | 9 | 138.9 | 0.017 |
| **GRIN1** | 9 | 139.2 | 0.017 |
| **ITSN1** | 21 | 33.9 | 0.0081 |
| **NRIP1** | 21 | 15.3 | 0.0026 |
| **CABIN1** | 22 | 22.7 | 0.010 |
| **SYNJ1** | 21 | 32.9 | 0.0068 |

Chr: chromosome

**Supplementary Table 5:** Pathways with most significant enrichment for ADHD case CNV hits among CNVs >500kb, showing enrichments in deletions and duplications.

| **Pathway Name** | **Pathway ID** | **# genes** | **p(all CNV)** | **p(del)** | **p(dup)** | **P (del vs dup)** |
| --- | --- | --- | --- | --- | --- | --- |
| IL6-mediated signalling events | NCI:62 | 82 | 1.33x10^-11^ | 0.25 | 4.65x10^-11^ | *0.017* |
| TGF-beta receptor signalling | NCI:95 | 44 | 4.90x10^-11^ | 0.93 | 5.21x10^-11^ | *1.13x10^-5^* |
| Defense response to virus | GO:51607 | 147 | 7.02x10^-8^ | 0.047 | 7.24x10^-7^ | 0.21 |
| **Respiratory electron transport** | **REACT:1019** | **81** | **1.98x10^-7^** | **0.15** | **3.02x10^-7^** | **0.60** |
| **Organonitrogen compound catabolic process** | **GO:1901565** | **893** | **9.08x10^-7^** | **0.21** | **6.09x10^-7^** | **0.11** |
| **Transmembrane transporter activity** | **GO:22857** | **902** | **9.10x10^-7^** | **0.020** | **6.30x10^-6^** | **0.23** |
| Citric acid (TCA) cycle and respiratory electron transport | REACT:1240 | 118 | 1.16x10^-6^ | 0.16 | 1.48x10^-6^ | 0.28 |
| **Carbohydrate derivative catabolic process** | **GO:1901136** | **747** | **2.19x10^-6^** | **0.054** | **9.69x10^-6^** | **0.049** |
| **Ligand-gated ion channel activity** | **GO:15276** | **136** | **2.33x10^-6^** | **0.007** | **6.09x10^-5^** | **0.67** |
| **Methyltransferase activity** | **GO:8168** | **201** | **3.19x10^-6^** | **0.22** | **1.38x10^-6^** | **0.27** |
| Small thymus | MGI:706 | 199 | 4.15x10^-6^ | 0.018 | 1.02x10^-4^ | 0.0087 |
| **Transmembrane transport** | **GO:55085** | **1124** | **5.22x10^-6^** | **0.032** | **2.38x10^-5^** | **0.23** |
| **Ion gated channel activity** | **GO:22839** | **300** | **5.29x10^-6^** | **0.0058** | **9.67x10^-5^** | **0.52** |

Pathways which are robustly enriched (i.e. those with p(minusbest)<0.05 in Table 4) are shown in bold. Pathways with stronger enrichment in duplications compared to deletions (i.e. those where case duplications are significantly more likely than case deletions to hit pathway genes) are shown in italics in the final column

**Supplementary Table 6:** The most significant pathways from the meta-analysis of CNVs >500kb (Table 4) are presented with the numbers of gene hits in cases and controls and the effect size (regression coefficient (coeff) from the logistic regression analysis of enrichment). The p-values, numbers of gene hits and effect size are also given when the sample giving the most significant enrichment is removed. Pathways for which the effect size in the most significant sample is significantly (p<0.05) different to that in the remaining samples are italicised.

| **Pathway Name** | **Pathway** | **Meta-analysis of all samples** | | | | **Meta-analysis excluding the most significant sample** | | | |
| --- | --- | --- | --- | --- | --- | --- | --- | --- | --- |
|  |  | **p** | **# hits in cases** | **# hits in controls** | **coeff** | **P** | **# hits in cases** | **# hits in controls** | **coeff** |
| *IL6-mediated signalling events* | *NCI:62* | *1.33x10^-11^* | *64* | *26* | *2.18* | *0.26* | *3* | *7* | *0.57* |
| TGF-beta receptor signalling | NCI:95 | 4.90x10^-11^ | 58 | 23 | 2.28 | 0.51 | 1 | 3 | -0.03 |
| *Defense response to virus* | *GO:51607* | *7.02x10^-8^* | *62* | *24* | *1.65* | *0.31* | *8* | *10* | *0.29* |
| Respiratory electron transport | REACT:1019 | 1.98x10^-7^ | 35 | 14 | 1.79 | 0.020 | 8 | 5 | 1.24 |
| Organonitrogen compound catabolic process | GO:1901565 | 9.08x10^-7^ | 219 | 274 | 0.71 | 3.13x10^-5^ | 187 | 260 | 0.64 |
| Transmembrane transporter activity | GO:22857 | 9.10x10^-7^ | 297 | 355 | 0.65 | 8.85x10^-4^ | 126 | 197 | 0.58 |
| Citric acid (TCA) cycle and respiratory electron transport | REACT:1240 | 1.16x10^-6^ | 38 | 19 | 1.53 | 0.057 | 9 | 8 | 0.84 |
| Carbohydrate derivative catabolic process | GO:1901136 | 2.19x10^-6^ | 179 | 218 | 0.73 | 3.34x10^-4^ | 73 | 110 | 0.78 |
| Ligand-gated ion channel activity | GO:15276 | 2.33x10^-6^ | 81 | 61 | 0.95 | 3.41x10^-4^ | 64 | 56 | 0.78 |
| Methyltransferase activity | GO:8168 | 3.19x10^-6^ | 81 | 67 | 0.97 | 0.022 | 26 | 31 | 0.64 |
| *Small thymus* | *MGI:706* | *4.15x10^-6^* | *64* | *51* | *1.1* | *0.49* | *13* | *28* | *0.01* |
| Transmembrane transport | GO:55085 | 5.22x10^-6^ | 334 | 417 | 0.58 | 0.0028 | 138 | 231 | 0.5 |
| Ion gated channel activity | GO:22839 | 5.29x10^-6^ | 125 | 109 | 0.82 | 4.18x10^-4^ | 44 | 56 | 0.84 |

**Supplementary Table 7: Top ion channel genes**

| **Gene** | **Chr** | **Position (Mb)** | **p(500kb)** |
| --- | --- | --- | --- |
| KCNJ6 | 21 | 37.9 | 0.0028 |
| GRIK1 | 21 | 29.8 | 0.0061 |
| KCNJ15 | 21 | 38.6 | 0.0066 |
| CLIC6 | 21 | 35 | 0.0078 |
| KCNE1 | 21 | 34.7 | 0.0078 |
| GABRG3 | 15 | 24.8 | 0.0083 |
| RYR3 | 15 | 31.4 | 0.010 |
| CHRNA7 | 15 | 30.1 | 0.015 |
| GRIN1 | 9 | 139.2 | 0.017 |
| CLIC3 | 9 | 139 | 0.017 |
| KCNE2 | 21 | 34.7 | 0.019 |

Chr: chromosome

**Supplementary Table 8:** Top catabolic process genes

| **Gene** | **Chr** | **Position (Mb)** | **p(500kb)** |
| --- | --- | --- | --- |
| CCT8 | 21 | 29.4 | 0.0026 |
| ATP5J | 21 | 26 | 0.0033 |
| GPC6 | 13 | 92.7 | 0.0070 |
| ATP5O | 21 | 34.2 | 0.0081 |
| UPB1 | 22 | 23.2 | 0.010 |
| CBS | 21 | 43.3 | 0.011 |
| GPC5 | 13 | 90.8 | 0.018 |
| GPT2 | 16 | 45.5 | 0.019 |
| ATP1B1 | 1 | 167.3 | 0.025 |
| ABCC6 | 16 | 16.2 | 0.028 |
| ATP10A | 15 | 23.5 | 0.029 |

Chr: chromosome

**Supplementary Table 9:** Top transmembrane transport genes

| **Gene** | **Chr** | **Position (Mb)** | **p(500kb)** |
| --- | --- | --- | --- |
| KCNJ6 | 21 | 37.9 | 0.0028 |
| ATP5J | 21 | 26 | 0.0033 |
| GRIK1 | 21 | 29.8 | 0.0061 |
| KCNJ15 | 21 | 38.6 | 0.0066 |
| CLIC6 | 21 | 35 | 0.0078 |
| KCNE1 | 21 | 34.7 | 0.0078 |
| ATP5O | 21 | 34.2 | 0.0081 |
| GABRG3 | 15 | 24.8 | 0.008 |
| RYR3 | 15 | 31.4 | 0.010 |
| SLC2A11 | 22 | 22.5 | 0.010 |
| SLC37A1 | 21 | 42.8 | 0.011 |
| CHRNA7 | 15 | 30.1 | 0.015 |
| GRIN1 | 9 | 139.2 | 0.017 |
| CLIC3 | 9 | 139 | 0.017 |

Chr: chromosome
